# Right lower lobectomy and lymph node dissection for lung cancer with subcarinal bronchial diverticulum

**DOI:** 10.1093/jscr/rjaf223

**Published:** 2025-04-19

**Authors:** Norichika Iga, Yohei Hayama, Eiji Yamada, Masahiko Muro

**Affiliations:** Department of Thoracic Surgery, Fukuyama City Hospital, 5-23-1 Zao-cho, Fukuyama 721-8511, Japan; Department of Thoracic Surgery, Fukuyama City Hospital, 5-23-1 Zao-cho, Fukuyama 721-8511, Japan; Department of Thoracic Surgery, Fukuyama City Hospital, 5-23-1 Zao-cho, Fukuyama 721-8511, Japan; Department of Thoracic Surgery, Fukuyama City Hospital, 5-23-1 Zao-cho, Fukuyama 721-8511, Japan

**Keywords:** bronchial diverticulum, lung cancer, robot-assisted thoracic surgery

## Abstract

A tracheobronchial diverticulum is an incidental airway abnormality often identified on chest computed tomography. Thoracic surgery involves manipulation around the bronchial diverticulum, assessing its presence, and implementing appropriate strategies to prevent injury. However, few reports document cases of thoracic surgery complicated by a bronchial diverticulum. Herein, we report a case of surgical resection of lung cancer with a bronchial diverticulum. A 66 year-old woman was diagnosed with right lower lobe lung cancer, c-T1b N1 M0 stage IIB right lower-lobe lung cancer. Chest computed tomography showed a 9 mm bronchial diverticulum below the carina. Surgical intervention for bronchial diverticulum was performed simultaneously with the dissection of the inferior mediastinal lymph nodes. Right lower lobectomy with lymph node resection was safely performed using robot-assisted surgery.

## Introduction

A bronchial diverticulum (BD) is an airway abnormality detected incidentally on chest computed tomography (CT). In thoracic surgery involving manipulation around the trachea and bronchi, evaluating the presence of bronchial diverticula (BDs) is important. Additionally, injury should be avoided due to the risk of respiratory management disorders and postoperative complications. However, only a few cases of thoracic surgery complicated with BD have been reported to date. Here, we present a case of right lower lobectomy for lung cancer with BD below the carina, in which surgical treatment for the diverticulum and mediastinal lymph node dissection was performed.

## Case report

**Figure 1:**
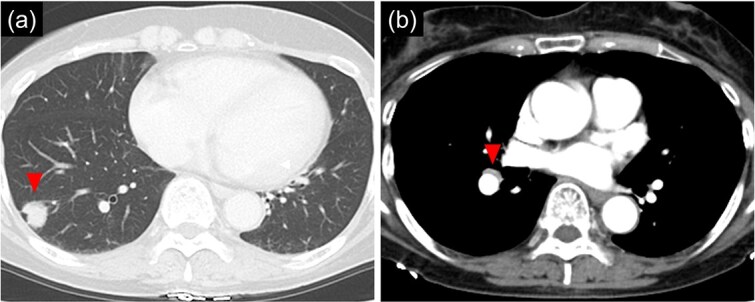
Chest computed tomographic image. (a) An 18 mm size nodule is detected in the S9 region of the right lower lobe. (b) An enlarged lymph node (#11iLN) with fluorodeoxyglucose accumulation is located anterior to the basal segment of the pulmonary artery.

**Figure 2:**
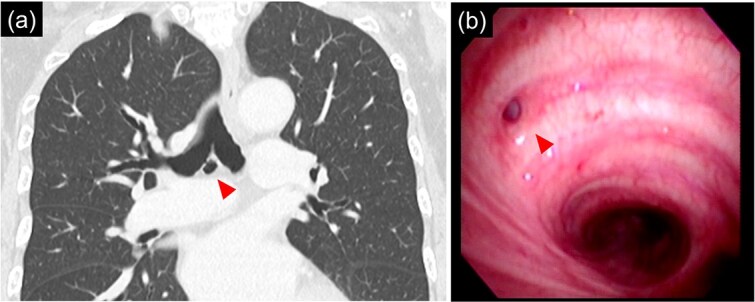
(a) 9 mm size bronchial diverticulum located at the subcarinal region. (b) Bronchial diverticulum opening in the right main bronchus and two rings distal to the carina.

The patient was a 66 year-old woman with no history of smoking or other medical history. Chest CT showed an 18 mm nodule suspected to be a primary lung cancer in the S9 region of the right lower lobe (Fig. 1). CT-guided lung biopsy revealed lung adenocarcinoma. PET/CT showed FDG accumulation in #11i lymph node suspicious for metastasis, and the preoperative staging was c-T1bN1M0 Stage IIB. The patient was referred to the Department of Thoracic Surgery in our hospital. In addition, a 9 mm size BD communicating with the right main bronchus was observed below the tracheal bifurcation in the preoperative image (Fig. 2). Right lower lobectomy and mediastinal lymph node dissection were planned; however, because the inferior mediastinal tissue was to be dissected, a procedure for BD was also considered necessary. To achieve safe dissection around the BD, robotic thoracoscopic surgery was planned, taking advantage of the fine movement and highly flexible forceps manipulation.

**Figure 3:**
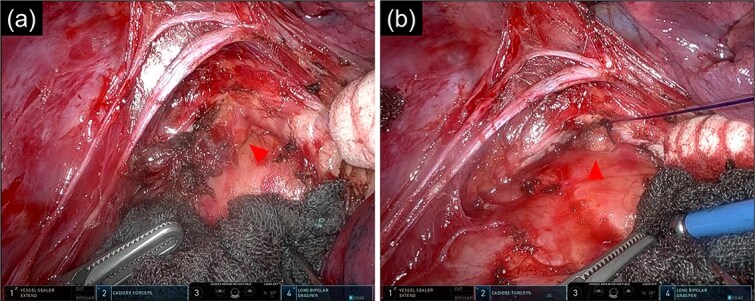
Intraoperative images of the inferior mediastinal region. (a) Image of dissecting the bronchial diverticulum and mediastinal lymph nodes. (b) Image of a bronchial diverticulum after ENDOLOOP® PDS II Ligature ligation. Arrowhead: bronchial diverticulum.

Bronchoscopy after intubation revealed an orifice of the BD in the cartilage of the right main bronchus with two rings below the carina (Fig. 2). First, a lower mediastinal lymph node dissection and BD were performed. The area around the BD was prone to bleeding and the lymph nodes were in close contact with each other (Fig. 3), making it difficult to separate them from the BD. After identifying and exposing the stalk of a BD, ligation of the diverticulum with ENDOLOOP® PDS II Ligature allowed for control of bleeding and the lymph nodes could be safely removed from the BD (Video 1).

After lymph node dissection was complete (Fig. 3), the BD was additionally ligated. The #11i LN was enlarged but without invasion, and there was no lymph node metastasis on frozen pathology. Following a conventional right lower lobectomy, the BD and lower lobe bronchial stumps were covered with pericardial fat tissue. The patient was discharged without postoperative complications. No lymph node metastases were found pathologically in the other dissected lymph nodes, and the diagnosis was p-T1bN0M0 stage IA2. Chest CT 6 months after the operation showed no recurrence of lung cancer; however, air was detected in the BD, suggesting an opening of the diverticulum.

## Discussion

A tracheobronchial diverticulum is an airway abnormality arising from the tracheal or bronchial wall that communicates directly with the lumen. The incidence of tracheal diverticulum is reported to be 2.4%–9.5% [1], and BDs are relatively common, found on chest CT in 33.9% of non-smokers (46.5% of men vs. 26.9% of women) and reported to be found in 45.5% of smokers [2, 3]. Although some articles have discussed the causes of congenital and acquired tracheal diverticula [4], little is known about the aetiology of BDs. Only one case of thoracic surgery involving oesophageal cancer with BD has been reported in the literature [1]. Performing a surgical procedure for BD detected without any symptoms, as in the present case, is controversial. There have been a few reports that the tracheal diverticulum is perforated by positive-pressure ventilation under general anaesthesia and caused pneumomediastinum [5, 6]. In lung cancer surgery, mediastinal lymph node dissection is necessary; the BD is exposed, and the mediastinal tissue opens into the thoracic cavity. Moreover, mediastinal lymph node dissection carries a risk of injury to the BD. Perforation of BD in the presence of the mediastinum that opens it into the thoracic cavity is expected to cause serious complications similar to those of a bronchial fistula. Therefore, we decided to perform a surgical procedure for the BD. Intraoperative bronchoscopy was useful for identifying the BD orifice because it was difficult to distinguish the stalk of the bronchial diverticulum from the thoracic cavity. Lymph nodes surround the peripheral region of the BD and bleed easily, making lymph node dissection difficult while preserving the diverticulum. The flexible movement of the robotic forceps helped reduce the burden of the surgical procedure. In addition, ligating the BD first when controlled oozing of the dissected tissue was recognized reduced the risk of perforation of the diverticulum wall, allowing the operation to be completed safely.

In the present case, a follow-up CT 6 months after the operation showed air in the diverticulum, suggesting an opening of the BD. The diverticulum was ligated using an ENDOLOOP® PDS II Ligature absorbable thread, and recanalization may have occurred due to hydrolysis of the absorbable thread. These findings suggest that nonabsorbable threads should be used for ligation of diverticulum.

## Conclusion

It is important to carefully evaluate preoperative images for the presence of BDs in thoracic surgery involving tracheal and peri bronchial manipulation because BDs are relatively common on Chest CT. In robot-assisted thoracoscopic surgery, mediastinal lymph node dissection and treatment of BD can be safely performed owing to the flexibility of the robotic forceps.

## Supplementary Material

Video_1_rjaf223

## Data Availability

The data underlying this article will be shared on reasonable request to the corresponding author.
